# The role and mechanisms of matrix metalloproteinase-9 in peripheral neuropathic pain

**DOI:** 10.3389/fnmol.2025.1647316

**Published:** 2025-10-30

**Authors:** Shuai Ying Jia, Xiao-Jun Tang, Yao Su, Yu-Ning Liu, Zhi Ming, Jing-Yan Lin

**Affiliations:** Department of Anesthesiology, The Affiliated Hospital of North Sichuan Medical College, Nanchong, China

**Keywords:** matrix metalloproteinase-9, peripheral neuropathic pain, nerve injury, pathophysiology, treatment strategy

## Abstract

Peripheral neuropathic pain is a chronic, secondary pain state caused by damage or diseases of the peripheral nervous system, typically accompanied by edema, inflammatory responses, increased neuronal excitability, and glutamate accumulation. Matrix metalloproteinase-9 (MMP-9), an important enzyme, plays a key role in various physiological and pathological processes, primarily by degrading the extracellular matrix. Recent studies have shown that MMP-9 plays a crucial role in the onset and progression of central nervous system disorders, particularly neuropathic pain. This review discusses the mechanisms underlying the involvement of MMP-9 in various models of peripheral neuropathic pain, with the aim of exploring its potential as a therapeutic target.

## 1 Introduction

Neuropathic pain is a complex condition that results from damage or dysfunction within the somatosensory nervous system. It affects about 7–10% of people globally and is particularly common among middle-aged individuals and women (van Hecke et al., 2014). There are two main types of neuropathic pain based on the underlying cause: central and peripheral. Central neuropathic pain arises from damage to the brain or spinal cord, while peripheral neuropathic pain is caused by injury to the peripheral nervous system (Scholz et al., 2019). Peripheral neuropathic pain is characterized by a wide range of clinical symptoms and a complex pathophysiology. These mechanisms are distinct from the pain caused by tissue damage or disease. Common symptoms include radicular pain, burning sensations, tingling, and numbness (Meacham et al., 2017). In addition to persistent pain, patients often experience sleep disturbances, depression, anxiety, and significant difficulties in daily life. These factors collectively reduce the overall quality of life for individuals suffering from the condition (Smith and Torrance, 2012). Furthermore, peripheral neuropathic pain often does not respond well to standard analgesic treatments, which highlights the urgent need for more effective therapeutic strategies (Finnerup et al., 2021).

Matrix metalloproteinase-9 (MMP-9), also known as gelatinase B, is a zinc-dependent endopeptidase in the matrix metalloproteinase family. MMP-9 regulates interactions between cells and the extracellular matrix by breaking down its components. This process influences cell migration, inflammation, proliferation, and apoptosis (Cabral-Pacheco et al., 2020). During inflammatory responses, MMP-9 plays a key role in the migration and activation of leukocytes. Recent studies have highlighted its crucial involvement in the development and progression of several neuropathic conditions, including trigeminal neuralgia, diabetic neuropathy, postherpetic neuralgia, and chronic neuropathic pain following peripheral nerve injury (Yin et al., 2019; Singh et al., 2020; Pan et al., 2018; Fan et al., 2018). Elevated MMP-9 activity is closely linked to post-injury inflammation, neuron–glia interactions, and pain signal transmission (Umbricht, 2022; Huang et al., 2021; Remacle et al., 2015). Consequently, MMP-9 has emerged as a key therapeutic target in neuropathic pain research.

**Figure 1:**
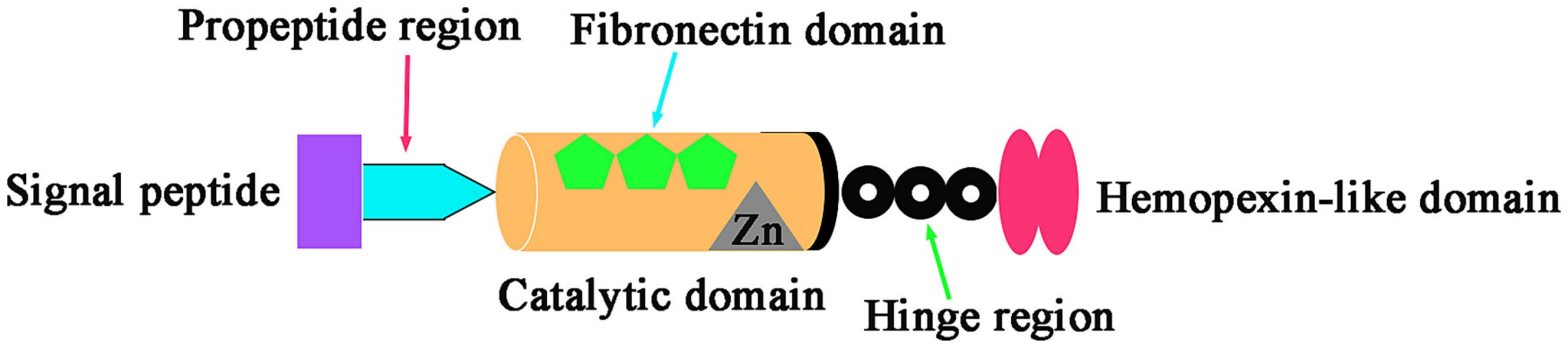
The structure of MMP9. This diagram shows the main structure of MMP9.

MMP-9 is encoded by the gelatinase B gene located on human chromosome 20. This gene consists of 13 exons and 12 introns. The protein encoded by MMP-9 has a complex structure, including a signal peptide, propeptide, hinge region, catalytic domain, and a hemopexin-like domain (Nagase et al., 2006). The catalytic domain contains a fibronectin type II domain, an active site, and a zinc-binding region that relies on zinc ions for its catalytic function. MMP-9’s primary role is the degradation of the extracellular matrix (ECM) (Agrawal et al., 2008). Its structure is shown in Figure 1. Additionally, MMP-9 is involved in important biological processes such as tissue remodeling, wound healing, skeletal development, and immune responses (Qorri et al., 2018; Hingorani et al., 2018; Malemud, 2006; Bratcher et al., 2012; McClellan et al., 2006).

The expression of MMP-9 is regulated at multiple levels, including gene transcription and post-translational modifications. At the transcriptional level, transcription factors like activator protein 1 (AP-1) and nuclear factor kappa-light-chain-enhancer of activated B cells (NF-κB) regulate MMP-9 expression in response to external stimuli such as growth factors, cytokines, and stress signals (Wang T. et al., 2018; Li et al., 2020; Murthy et al., 2010). Epigenetic modifications, including DNA methylation and histone acetylation, also play a significant role in regulating MMP-9 expression (Duraisamy et al., 2017; Yeh et al., 2016). At the post-translational level, MMP-9 is secreted as an inactive proenzyme (Pro-MMP-9) and is activated through cleavage by specific proteases. Membrane-type matrix metalloproteinase removes the inhibitory sequence from the proenzyme, thus activating its catalytic function (Dreier et al., 2004). Furthermore, the activity of MMP-9 is tightly controlled by tissue inhibitors of metalloproteinases (TIMPs), which bind to MMP-9 and prevent its interaction with substrates. This regulation is critical for maintaining the integrity of the extracellular matrix and preventing excessive degradation (Filanti et al., 2000).

## 2 Role of MMP-9 in different types of peripheral neuropathic pain

According to the latest classification system by the International Association for the Study of Pain (IASP), this paper reviews several common subtypes of peripheral neuropathic pain. These include trigeminal neuralgia, chronic neuropathic pain after peripheral nerve injury, painful polyneuropathy, postherpetic neuralgia, and painful radiculopathy (Treede et al., 2019).

### 2.1 Trigeminal neuralgia

Trigeminal neuralgia (TN) is a well-known condition characterized by severe pain along the trigeminal nerve. It is typically categorized into three subtypes based on its cause: classical, secondary, and idiopathic (Anonymous, 2018). The main symptom of TN is sudden, sharp facial pain on one side of the face, often affecting one or more branches of the trigeminal nerve (Edlich et al., 2006). Patients often describe the pain as sharp, electric shock-like, or stabbing. Episodes can last from a few seconds to a few minutes. Pain is commonly triggered by mild stimuli such as light facial touch, chewing, or even exposure to wind. In some cases, patients may also experience involuntary facial muscle contractions or spasms (Jones et al., 2019; Bendtsen et al., 2020). Epidemiological studies report an annual incidence of TN ranging from 4.3 to 27 cases per 100,000 individuals. It is more common in people over 60 years old and more prevalent in women, with an incidence rate of 5.9 per 100,000 compared to 3.4 per 100,000 in men (Shaefer et al., 2018).

In classical trigeminal neuralgia, the vascular compression hypothesis is the most widely accepted explanation (Anonymous, 2018). Approximately 50% of cases are attributed to compression by the superior cerebellar artery, while about 25% are caused by venous compression (Hamlyn and King, 1992). Vascular compression, often due to abnormal dilation of intracranial arteries or veins, leads to demyelination of the trigeminal nerve root. This results in abnormal nerve signaling and pain (Marinković et al., 2009). Moreover, the abnormal activation of specific sodium and potassium channels on nerve membranes contributes to neuronal hyperexcitability and ectopic discharges, key mechanisms in the pathophysiology of TN (Liu et al., 2019; Ling et al., 2018). Secondary trigeminal neuralgia is usually associated with facial trauma or surgeries involving the trigeminal nerve or its branches (Renton et al., 2010). Other factors, such as genetic predisposition, infections like postherpetic neuralgia, and autoimmune disorders, can impair trigeminal nerve function and trigger or worsen pain (Fernández Rodríguez et al., 2019; Wang et al., 2023; Ferreira et al., 2020).

The main treatment for TN is pharmacological, with carbamazepine and other anticonvulsants being the first-line options. Botulinum toxin and local anesthetics have also been found to provide quick and effective pain relief (Kowacs et al., 2015; Han et al., 2008). More recently, novel sodium channel blockers have shown promising results in clinical trials (Zakrzewska et al., 2017; Dunbar et al., 2020). For patients who do not respond to medication, surgical options such as microvascular decompression and nerve block procedures can be effective and may be considered either as first-line or secondary treatments following recurrence (Xia et al., 2014; Wang et al., 2022).

Recent studies using chronic constriction injury (CCI) models in CD-1 mice have shown that MMP-9 and MMP-2 expression increases significantly in the trigeminal ganglion at different time points. This suggests that MMP-9, in particular, may play a crucial role in the onset and progression of trigeminal neuralgia (Yin et al., 2019). In models of spinal nerve ligation (SNL) and infraorbital nerve CCI (CCI-IoN), blocking MMP-9/2 activity significantly reduces mechanical allodynia. This indicates that targeting MMP-9/2 could be a promising strategy for pain relief (Henry et al., 2015). Similarly, in a rat model of temporomandibular joint arthritis, mechanical allodynia of the trigeminal nerve was observed. This was accompanied by increased MMP-9 expression and activity in both the limbic system and trigeminal ganglion. These changes may be linked to a reduction in immunoreactivity of the voltage-gated K + channel subtype 1.4 in trigeminal ganglion neurons (Takeda et al., 2008; Nascimento et al., 2021). Low-level laser therapy (LLLT) has been shown to reduce MMP-9 levels in the trigeminal ganglion, decrease its gelatinolytic activity, and alleviate both mechanical allodynia and orofacial hyperalgesia. These results suggest that LLLT may aid in tissue repair and limit extracellular matrix degradation (Desiderá et al., 2015; Lemos et al., 2016). Taken together, these studies underline the central role of MMP-9 in the development of trigeminal neuralgia and provide a strong foundation for exploring new treatment options, with MMP-9 inhibition emerging as a particularly promising approach.

### 2.2 Neuropathic pain following peripheral nerve injury

Peripheral nerve injury causes both structural damage and functional impairments, often accompanied by significant neuropathic pain (Lopes et al., 2022). In response to injury, the affected nerve region rapidly triggers an inflammatory reaction. This reaction recruits immune cells, including macrophages, neutrophils, and T-cells (Balog et al., 2023; Moalem et al., 2004). These immune cells release proinflammatory mediators such as tumor necrosis factor-alpha (TNF-*α*), interleukin-1 beta (IL-1β), interleukin-6 (IL-6), and prostaglandins. These mediators amplify inflammation and increase nerve sensitivity. As a result, pain intensity increases and persists (Nadeau et al., 2011; Gui et al., 2016).

Recent studies have shown that MMP-9 plays a key role in the development of neuropathic pain. MMP-9 influences the function of the voltage-gated sodium channel subunit Nav1.7, which contributes to pain onset. It also interacts with N-methyl-D-aspartate receptors (NMDARs), which regulate synaptic plasticity and memory formation (Remacle et al., 2015; Wiera et al., 2017). Specifically, during neurodevelopment, MMP-9 accelerates the functional inactivation of the Nav1.7 sodium channel by hydrolyzing its external exposed region, particularly the structural region between the S5-S6 transmembrane segments. MMP-9 may also affect neuronal conduction by modulating the extracellular matrix and relevant signaling pathways. For instance, MMP-9 might influence local neuronal conduction properties through its actions on the extracellular matrix. Additionally, MMP-9 activity may interact with inflammatory response pathways, such as the NF-κB pathway. This interaction further exacerbates plasticity changes in the nervous system, contributing to pain regulation and other neurodegenerative diseases (Hackel et al., 2012; Remacle et al., 2015; Wang et al., 2019). In various pain models, MMP-9 has been shown to influence NMDA receptors. For example, in the bone cancer pain model, the upregulation of MMP-9 activates Ephrin type-B receptor 1. This activation enhances the phosphorylation of NMDA receptor subunits NR1 and NR2B, leading to increased Ca^2+^ influx. This amplifies downstream signaling, promoting hyperalgesia and opioid tolerance (Liu et al., 2011). In a mouse plantar incision model, MMP-9 activity significantly increases in the spinal cord and glial cells. This activity contributes to postoperative hyperalgesia through the p38/IL-1β signaling pathway (Li et al., 2023; Jiang et al., 2020; Gu et al., 2019). Similarly, during the early stages of chronic sciatic nerve constriction injury in rats, MMP-9 activity is strongly associated with elevated levels of the chemokine C-X3-C motif ligand 1 (CX3CL1) (Zhao et al., 2020). In the sciatic nerve crush (SNC) rat model, overexpression of MMP-9 is linked to excessive activation of the TRPV1 channel, intensifying pain perception and inflammatory responses (Awad-Igbaria et al., 2023). Inhibition of MMP-9 activity or expression has been shown to effectively alleviate both postoperative and neuropathic pain (Lv et al., 2018; Liu et al., 2017). These findings emphasize the central role of MMP-9 in nerve injury and pain progression. Further research into MMP-9-mediated mechanisms may offer valuable insights into the molecular basis of pain and pave the way for developing novel therapeutic strategies.

### 2.3 Painful polyneuropathy

Painful polyneuropathy is generally classified into two main types: diabetic and non-diabetic (Scholz et al., 2019). Among these, painful diabetic neuropathy (PDN) is the most common, affecting about 20–30% of people with diabetes (Abbott et al., 2011; Aronson et al., 2021). Patients with PDN often report sharp, electric shock-like pain in their feet. This pain can become chronic and significantly lower their quality of life. In many cases, PDN is accompanied by psychological issues such as anxiety, depression, and sleep problems (Gylfadottir et al., 2020). Although the exact biological mechanisms behind PDN remain unclear, treatment primarily focuses on symptom relief. Common drugs include tricyclic antidepressants (TCAs), duloxetine, pregabalin, and gabapentin (Javed et al., 2015; Iqbal et al., 2018). However, these medications often cause side effects like nausea, drowsiness, and constipation. These adverse effects can reduce patient compliance and overall satisfaction with treatment (Tesfaye et al., 2022). Among non-diabetic causes of painful polyneuropathy, chemotherapy-induced peripheral neuropathy (CIPN) is especially common. It usually develops after treatment with chemotherapy agents such as paclitaxel or cisplatin. Patients with CIPN often experience persistent pain, numbness, and increased sensitivity to temperature or touch. In severe cases, these symptoms greatly impact daily functioning and quality of life (Hu et al., 2019).

PDN involves a complex interplay of multiple biological factors. Research has shown the involvement of pro-inflammatory cytokines (such as TNF-*α* and IL-1*β*), ion channels in sensory neurons, T-type calcium channels, as well as microglia and astrocytes (Yamakawa et al., 2011; Hangping et al., 2020; Bierhaus et al., 2012; Orestes et al., 2013; Liao et al., 2011). Additionally, the accumulation of harmful metabolic byproducts—such as reactive oxygen species (ROS), inflammatory transcription factors, and glutamate—can further drive the progression of PDN (Rivera-Aponte et al., 2015; Rendra et al., 2019). Due to the incomplete understanding of these mechanisms, no standardized treatment for PDN has been established. CIPN shares many of the same biological triggers. These include damage to intraepidermal nerve fibers (IENF), oxidative stress, ion channel activation, cytokine upregulation, and neuroimmune responses. Current treatments mainly rely on anti-inflammatory and pain-relief strategies to ease symptoms and improve patient comfort (Koskinen et al., 2011; Butturini et al., 2013; Zhang and Dougherty, 2014).

In preclinical studies, diabetic neuropathy models induced by streptozotocin in rats show heightened pain sensitivity and altered pain signaling. These models are widely used to explore PDN mechanisms. Treatments targeting oxidative-nitrosative stress, inflammatory cytokines, and MMP-9 activity have demonstrated significant reductions in neuropathic symptoms (Singh et al., 2020). More recent findings have highlighted a novel role for MMP-9 in cleaving beta-dystroglycan (β-DG). Inhibiting MMP-9 also affects the expression and localization of aquaporin-4 (AQP4) in the spinal cord’s glymphatic system. This modulation may enhance the clearance of metabolic waste in the central nervous system and offer a new therapeutic pathway for PDN (Li et al., 2024). Furthermore, network pharmacology studies have identified MMP-9 as a potential molecular target of Dolastatin 16, a compound involved in the TNF signaling pathway. Dolastatin 16 is associated with both diabetic foot ulcers and PDN. As a potential MMP-9 inhibitor, it may also promote wound healing in diabetic patients (Luthfiana and Utomo, 2023). Together, these findings suggest that MMP-9 is not only involved in the pathophysiology of PDN but also represents a promising target for future treatment strategies.

Recent clinical and laboratory studies have also drawn attention to the role of MMP-9 in CIPN. In patients receiving chemotherapy, higher levels of MMP-9 and high-mobility group box 1 (HMGB1) in the blood have been linked to more severe neuropathic symptoms (Yang et al., 2023). In animal models, increased MMP-9 expression in dorsal root ganglion (DRG) neurons has been associated with pain sensitivity, oxidative damage, and inflammation. Mechanistically, MMP-9 contributes to extracellular matrix remodeling, promotes neuroinflammation, and worsens neuronal injury through pathways like the HMGB1–TLR4–PI3K/Akt axis (Gu et al., 2020). Blocking MMP-9—either through gene knockout or pharmacological inhibition—has been shown to reduce CIPN severity in animal models. This evidence points to MMP-9 as a viable therapeutic target. Several strategies to inhibit MMP-9 have shown promise. These include monoclonal antibodies targeting MMP-9 and small-molecule inhibitors like N-acetylcysteine (NAC), which have successfully reduced symptoms in preclinical models (Tonello et al., 2019). In addition, chemotherapy drugs such as cisplatin can induce cellular senescence in neurons. This senescence process is often marked by increased MMP-9 expression and the release of inflammatory molecules—a phenomenon known as the senescence-associated secretory phenotype (SASP) (Acklin et al., 2020; Saleh et al., 2024). Altogether, these results underline the central role of MMP-9 in both the onset and progression of CIPN. Targeting MMP-9 could open new avenues for improving outcomes in cancer patients affected by this challenging condition.

### 2.4 Painful radiculopathy

Painful radiculopathy is typically caused by lesions in the cervical, thoracic, lumbar, or sacral nerve roots. Low back pain is the most common symptom associated with this condition(Scholz et al., 2019). Intervertebral disk herniation is a leading cause of low back pain, affecting more than 70% of individuals and often worsening with age (Hoy et al., 2010). In addition to severe discomfort, low back pain can limit daily activities and lead to occupational disability. This imposes a significant burden on patients, their families, and society (Will et al., 2018). While many patients with acute low back pain recover without long-term symptoms or functional impairment through conservative treatments, such as education, pharmacological therapy, and physical rehabilitation, about 30% experience a recurrence of pain within 1 year. In some cases, this can progress to chronic low back pain (da Silva et al., 2017).

The underlying mechanisms of chronic low back pain remain debated. It is generally believed that its onset is closely linked to mechanical compression of nerve roots due to intervertebral disk herniation, accompanied by local inflammatory responses (Erbüyün et al., 2018). Chronic low back pain is often triggered by a combination of neuropathic and nociceptive pain mechanisms (Freynhagen et al., 2006; Kaki et al., 2005). Furthermore, intervertebral disk degeneration is considered a key factor contributing to low back pain (Peng, 2013). As individuals age, cellular senescence and phenotypic changes, alongside dysfunction of the extracellular matrix (ECM), lead to early degenerative changes in the intervertebral disks. These changes, in turn, trigger inflammatory responses that contribute to the progression of pain (Bermudez-Lekerika et al., 2022; Lyu et al., 2021).

Current clinical guidelines recommend maintaining physical activity and engaging in regular exercise to manage chronic low back pain. Analgesics can be used when necessary. Nonsteroidal anti-inflammatory drugs (NSAIDs) are among the most commonly prescribed treatments. Short-term use of NSAIDs has been shown to alleviate pain effectively (Piccoliori et al., 2013). However, despite their analgesic and anti-inflammatory effects, NSAIDs can cause gastrointestinal and cardiovascular side effects (Sostres et al., 2013; Wehling, 2014). Because chronic low back pain often involves both neuropathic and nociceptive components, combining different types of analgesics is a rational approach to treatment. Personalized combination therapy can improve analgesic efficacy while minimizing the risk of side effects by reducing the dosages of individual medications (Müller-Schwefe et al., 2017).

For patients with chronic low back pain due to identifiable causes, such as nerve root compression from intervertebral disk herniation or spinal stenosis, surgery may be considered, especially in cases of severe pain. Lumbar fusion surgery has been shown to significantly reduce pain and improve functional outcomes (Helm Ii et al., 2012; Trescot et al., 2007). Some studies suggest that lumbar fusion surgery may influence MMP-9 activity by altering the mechanical load on the intervertebral disks or modulating the local inflammatory environment (Omair et al., 2013; Zhang et al., 2018). However, despite various surgical options, a thorough preoperative evaluation is essential (Chopko et al., 2014). Recent evidence suggests that long-term outcomes of surgical and non-surgical treatments are similar, highlighting the importance of carefully weighing the risks and high costs associated with spinal surgery (Mannion et al., 2013). Therefore, a comprehensive multidisciplinary rehabilitation program, combining pharmacological treatment, physical rehabilitation, psychological support, and addressing social and occupational factors, is recommended for optimal outcomes in patients with chronic low back pain (Guzmán et al., 2001).

**Figure 2:**
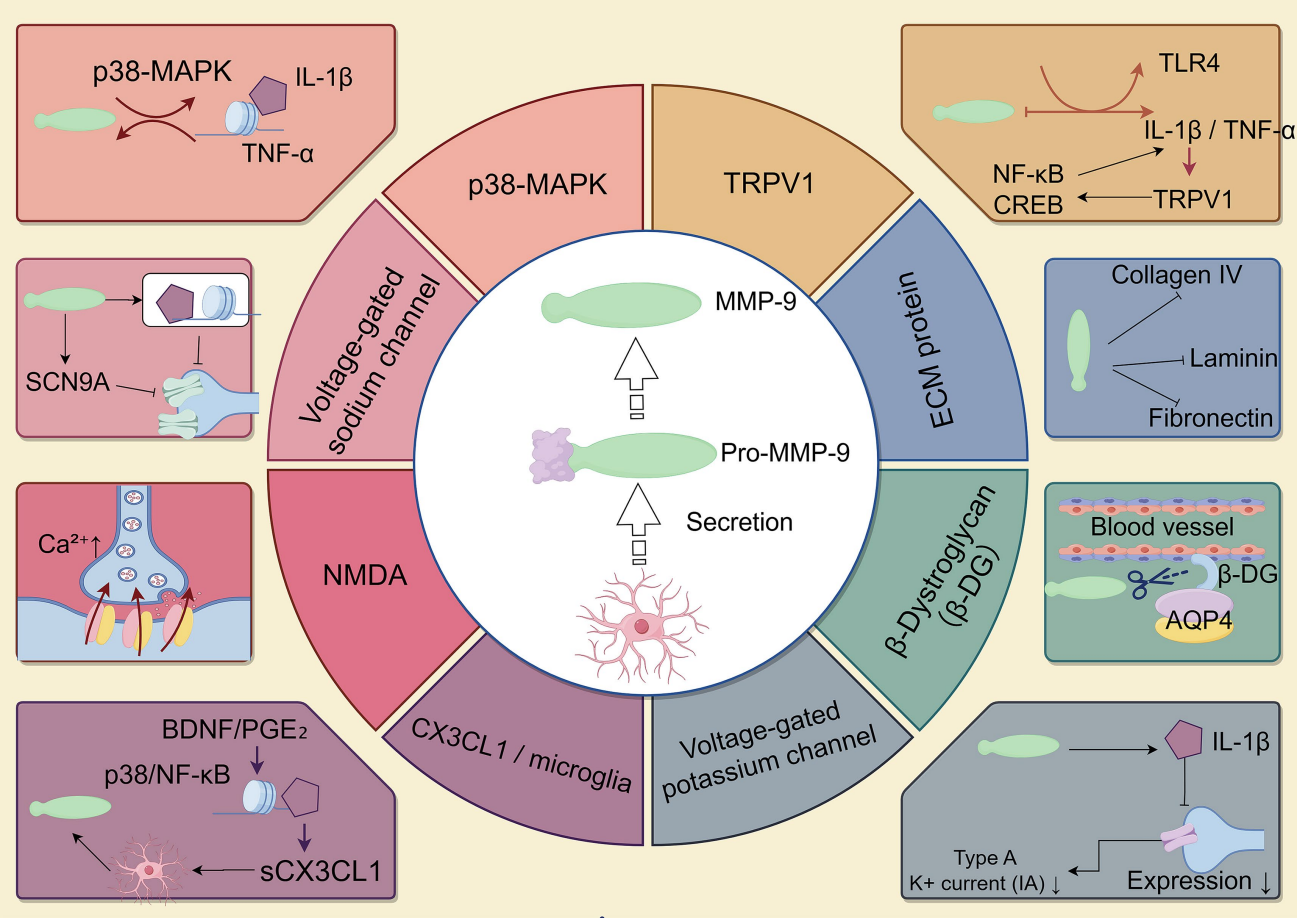
The Role and Mechanisms of Matrix Metalloproteinase-9 in Peripheral Neuropathic Pain. MMP9 plays distinct roles in different types of peripheral neuropathic pain, particularly in conditions such as trigeminal neuralgia, neuropathic pain following peripheral nerve injury, painful polyneuropathy, and painful radiculopathy. This diagram highlights its crucial involvement in the pathophysiological processes of these disorders, underscoring its key role in their pathogenesis.

Intervertebral disk degeneration (IDD) is characterized by cellular loss, ECM degradation, and reduced spinal flexibility (Yabe et al., 2015). Previous studies suggest that slowing ECM degradation may help delay disk degeneration (Gao et al., 2024). MMP-9, a key enzyme involved in ECM degradation, plays an important role in IDD, making it a potential therapeutic target. Research has shown that MMP-9 expression is upregulated in degenerated intervertebral disks (Wang W. J. et al., 2018). Inhibition of MMP-9 expression—via siRNA-mediated gene silencing, small molecule inhibitors (e.g., SB-3CT), or modulation through upstream signaling pathways such as miR-21/PTEN/Akt/mTOR—has been shown to reduce ECM breakdown and inflammatory responses *in vitro* NP cell models and *in vivo* rat models of IDD (Li et al., 2017; Gong et al., 2023; Shin et al., 2016). These findings suggest that controlling MMP-9 activity may slow the progression of disk degeneration. Additionally, genetic polymorphisms of MMP-9 have been identified as independent predictive factors for chronic low back pain and may influence patient recovery (Bjorland et al., 2019; Bjorland et al., 2017). MMP-9 also plays a significant role in low back pain associated with pregnancy, likely due to changes in the pelvic ligament ECM components caused by the rs17576 A > G polymorphism in the MMP-9 gene (Mahmood et al., 2018). While MMP-9 shows potential as a therapeutic target, its clinical feasibility is still under investigation. The development of selective MMP-9 inhibitors suitable for clinical use has been limited by issues such as off-target effects and poor bioavailability. Further studies using animal models and clinical samples are needed to assess the long-term efficacy and safety of these inhibitors (Kamieniak et al., 2019) (Table 1; Figure 2).

**Table 1:** The role of MMP-9 in different peripheral neuropathic pain models (preclinical studies).

Neuropathic pain model	MMP-9 expression	Mechanisms involved	Key findings	Key references
Chronic constriction injury of trigeminal nerve (mice)	MMP-9 was significantly upregulated in the trigeminal ganglion at early stages after CCI.	The TLR-4/NF-κB signaling pathway and SOCS3-mediated regulation were implicated in MMP-9/2 activation and pain sensitization.	Resveratrol pretreatment reduced MMP-9/2 activation and inflammatory cytokines	Yin et al. (2019)
Spinal nerve ligation model; chronic constriction injury of infraorbital nerve (rat)	Implicated indirectly via effect of MMP-2/−9 inhibition; not directly measured	Inhibition of MMP-2 and MMP-9 was shown to reduce mechanical allodynia	AQU-118, an MMP-2/9 inhibitor, demonstrated comparable efficacy to gabapentin	Henry et al. (2015)
Spinal nerve ligation (mice)	MMP-9 upregulated in spinal cord and DRG during neuroinflammation; Naru-3 reduces its expression	MMP-9/IL-1β signaling pathway-mediated microglia activation and inflammatory loop via p38/IL-1β axis	MMP-9 and IL-1β are key therapeutic targets of Naru-3’s synergistic analgesic effects	Zhou et al. (2023)
Sciatic nerve crush (rat)	MMP-9 expression increased after sciatic nerve crush; significantly reduced by Hyperbaric oxygen therapy in DRG and spinal cord	MMP-9 involved in inflammatory response; Hyperbaric oxygen therapy reduces MMP-9, TRPV1, cytokines (TNFα, IL-6, IL-1β), and mitochondrial stress (TSPO)	Hyperbaric oxygen therapy modulates inflammation and mitochondrial function, reduces neuronal apoptosis, and prevents progression from acute to chronic neuropathic pain	Awad-Igbaria et al. (2023)
Chronic constriction injury (rat)	Upregulated rapidly (as early as 3 h post-CCI)	MMP-9 and MMP-2 mediate TNF-α activation and receptor modulation, contributing to axonal degeneration and endoneurial inflammation.	Targeting MMP-9 and MMP-2 may help prevent TNF-α-mediated neuroinflammation and pain in neuropathic conditions.	Shubayev and Myers (2000)
Chronic constriction injury (rat)	MMP-9 expression was significantly upregulated by CCI and downregulated following GDNF treatment.	GDNF mitigates neural injury by inhibiting microglial activation and inflammatory cytokines via p38 and PKC signaling pathways.	GDNF shows anti-inflammatory and neuroprotective effects, making it a potential therapeutic option for CCI-induced neuropathic pain.	Chou et al. (2014)
Partial sciatic nerve ligation (mice)	Upregulated after PSNL; suppressed by anti-HMGB1 antibody	HMGB1 upregulates MMP-9 in injured nerve; both contribute to mechanical hypersensitivity	Blocking HMGB1 or inhibiting MMP-9 reduces neuropathic pain	Zhang et al. (2016)
Painful diabetic neuropathy (rat)	MMP-9 expression was significantly increased in PDN rats	Inhibiting the expression of MMP9 can alleviate PDN	MMP-9 is a potential therapeutic target for improving PDN	Li et al. (2024)

## 3 Discussion

Peripheral neuropathic pain places a heavy burden on both the body and mind. While current treatments can relieve some symptoms, they often fall short due to limited effectiveness, drug resistance, or side effects. As a result, there is an urgent need to uncover new molecular mechanisms and develop more targeted therapies. Among the molecules involved, matrix metalloproteinase-9 (MMP-9) has drawn increasing attention over the past decades. It is known for its key role in neuroinflammation and nerve remodeling in neuropathic pain.

MMP-9 contributes to pain progression by degrading extracellular matrix (ECM) components. This breakdown facilitates the infiltration of inflammatory cells and enhances its own expression, creating a feedback loop that worsens pain (Adams et al., 2015; Kwan et al., 2019). In addition, MMP-9 influences neuron–glia interactions and promotes nerve regeneration and axon growth, which are critical for recovery after nerve injury (Kim et al., 2012; Lu et al., 2022). It also indirectly affects how pain signals are transmitted by regulating cytokines and chemokines. For example, MMP-9 can degrade nerve growth factor (NGF) released by glial cells, which in turn affects pain sensitivity (Osikowicz et al., 2013). Furthermore, MMP-9 increases the permeability of the blood–brain barrier, allowing inflammatory molecules to enter the central nervous system and activate central pain pathways (Wang et al., 2024). The role of MMP-9 differs across pain types. In mechanical allodynia, it activates the NLRP3 inflammasome, which promotes the release of IL-1*β* and triggers a strong inflammatory response in the central nervous system (Deng et al., 2021). An imbalance between MMP-9 and its inhibitor TIMP1 has also been linked to pain. Disruption of the EZH2/TIMP1-MMP9 axis can activate the NLRP3 inflammasome and increase IL-1β secretion, further amplifying pain, especially in chronic constriction injury (CCI) models (Wan et al., 2025). MMP-9 also alters pain signaling by disrupting neuroglial interactions, breaking down neurotrophic factors such as NGF, and regulating ion channels like TRPV1 or transcription factors such as E74-like factor 1 (Li et al., 2023; Zhang et al., 2023). In thermal hyperalgesia, its involvement in nerve remodeling and pathways like PKC signaling appears more pronounced (Moon et al., 2012; Kim et al., 2014). Overall, higher levels of MMP-9 are often associated with more severe pain. Blocking MMP-9 activity has been shown to relieve symptoms in multiple experimental models, making it a promising therapeutic target (Table 2).

**Table 2:** Summary of major clinical studies.

Population	Intervention	Study design	Key findings	Limitations	Key references
14 healthy participants	Acupuncture was applied at specific points used in the treatment of spinal cord injury.	interventional study	Acupuncture significantly decreased BDNF and MMP-9 levels in peripheral blood.	small sample size and lack of clinical outcomes related to spinal cord injury.	Moldenhauer et al. (2010)
21 patients with Guillain-Barré syndrome	Plasma MMP-9 levels	observational correlation study	Higher plasma MMP-9 levels were associated with demyelination and peripheral nerve dysfunction in Guillain-Barré syndrome.	small sample size and lack of causal inference.	Sharshar et al. (2002)
86 patients with refractory diabetic dermal ulcers	Patients received either standard wound care or standard care plus topical application of autologous platelet-rich gel (APG).	randomized controlled trial	Topical APG significantly reduced ulcer area and improved proteolytic balance by lowering MMPs and increasing TIMP-1.	lacks long-term follow-up and detailed analysis of systemic effects.	He et al. (2012)
33 patients with chronic diabetic foot lesions (UT stage 2a)	Patients received either standard wound care or standard care plus daily application of ORC/collagen protease-inhibiting matrix.	interventional study	The ORC/collagen matrix reduced the MMP-9/TIMP-2 ratio	small sample size and short follow-up duration limited to 8 days	Lobmann et al. (2006)
12 patients with non-healing diabetic foot ulcers	Participants were randomized to receive non-contact low-frequency ultrasound (NCLF-US) either three times or once per week, or no ultrasound treatment.	prospective randomized clinical trial	Thrice-weekly NCLF-US treatment significantly reduced wound area and was associated with decreased MMP-9, pro-inflammatory cytokines and improved healing.	small sample size, limiting the generalizability of the results	Yao et al. (2014)
24 patients with diabetic foot ulcers	Topical propolis was applied to diabetic foot ulcers	Controlled Clinical Trial	Topical propolis significantly improved ulcer healing rates and reduced bacterial load and MMP-9 activity compared to controls.	small sample size, single-center design, and use of a non-randomized control group.	Henshaw et al. (2014)

The function of MMP-9 varies significantly between pain models. This may be due to its presence in different cell types, such as neurons and immune cells, and its interaction with specific ECM components (Folgueras et al., 2009; Martins-Oliveira et al., 2009). These differences suggest that treatments targeting MMP-9 should be tailored to the specific form of neuropathic pain. For example, in trigeminal neuralgia, which is often caused by vascular compression, the inflammatory role of MMP-9 may be more relevant (Henry et al., 2015). In contrast, in long-term neuropathic pain, its impact on nerve remodeling and regeneration becomes more important (Tonello et al., 2019). Optimizing drug development based on MMP-9’s specific function in each condition is essential. Evidence from animal studies shows that reducing MMP-9 activity can ease pain-related behaviors and hyperalgesia after nerve injury (Chattopadhyay et al., 2007; Zhang et al., 2016). Several inhibitors of MMP-9, such as BB-1101 and SB-3CT, have shown strong results in preclinical models. They reduce inflammation and protect neurons, contributing to pain relief (Demestre et al., 2004; Yin et al., 2019). Recent small-scale clinical studies echo these findings. For instance, Chu et al. (2020) found that patients with greater reductions in plasma MMP-9 levels after video-assisted thoracoscopic surgery reported less postoperative pain. This clinical observation supports the theory that MMP-9 plays a key role in pain regulation. Likewise, Ferianec et al. (2020) reported higher MMP-9 levels in patients with chronic pain, suggesting a link between circulating MMP-9 and pain severity. Taken together, both experimental and clinical data highlight the importance of MMP-9 in neuropathic pain. Targeting its activity could offer a new direction for developing effective and personalized pain therapies (Ferianec et al., 2020; Chu et al., 2020).

Recent studies have highlighted the role of MMP-9 in peripheral neuropathic pain. MMP-9 inhibitors are now under investigation as potential therapies. Several naturally derived compounds, such as β-sitosterol, resveratrol, mangiferin, and epigallocatechin-3-gallate, have shown analgesic effects in experimental models (Mohajeri et al., 2020; Lin et al., 2019; Tohge and Fernie, 2017; Yiemwattana et al., 2019; Subedi and Gaire, 2021). These agents also show promise in treating inflammatory and degenerative conditions. However, many of them are not selective for MMP-9. Their pain-relieving effects likely involve multiple mechanisms, including antioxidant and anti-inflammatory actions. Thus, the observed benefits cannot be attributed to MMP-9 inhibition alone.

Although MMP-9 is an attractive therapeutic target, its clinical application remains challenging. It interacts with inflammatory mediators such as IL-1β, TNF-*α*, and NMDA receptors, placing it at the center of neuropathic pain regulation (Berta et al., 2012). A deeper understanding of these interactions is needed to develop effective combination strategies that reduce the drawbacks of monotherapy. Another challenge comes from the dual role of MMP-9 in nerve repair. After peripheral nerve injury, MMP-9 supports regeneration by regulating communication between neurons and glial cells (Kawasaki et al., 2008). Excessive inhibition can ease pain, but over-inhibition may block repair (LeBert et al., 2015). Therefore, strategies must strike a balance: suppressing pain without halting neural recovery. MMP-9 also influences sodium channels, such as Nav1.7, and receptors including TRPV1, which play major roles in neuropathic pain (Remacle et al., 2015; Awad-Igbaria et al., 2023). E During neural repair, these interactions may be critical for pain development (Kim et al., 2012). This indicates that combining MMP-9 inhibitors with agents targeting sodium channels, NMDA receptors, or TRPV1 may improve efficacy and lower drug resistance. Still, most evidence so far comes from rodent models. Differences in expression patterns, immune responses, and pain phenotypes between species limit translation to humans. In addition, many studies rely on broad pharmacological inhibitors that carry off-target effects or trigger compensatory activation of other MMPs, such as MMP-2. These issues point to the need for selective genetic and proteomic approaches to define MMP-9’s precise role.

Clinical research will also be critical. Current preclinical findings show that MMP-9 inhibitors can reduce pain in animal models (Kwan et al., 2019). However, our study has limitations. Because we used non-selective compounds and broad-spectrum inhibitors, we cannot conclude that MMP-9 alone drives the observed effects. Future studies should use more selective tools—such as monoclonal antibodies, gene knockouts, or highly specific inhibitors—to clarify its exact contribution. Functional studies with these tools would also help minimize off-target concerns. Past failures of MMP inhibitors in clinical cancer trials remind us of the risks. These drugs lacked specificity, produced unacceptable side effects, and disrupted the function of MMPs with protective roles (Vandenbroucke and Libert, 2014). To avoid repeating this pattern, future success will depend on developing selective inhibitors that target disease-relevant MMPs like MMP-9, while sparing beneficial ones. Combining MMP-9 inhibition with agents targeting sodium channels, NMDA receptors, or TRPV1 may also offer synergistic benefits. In addition, advanced delivery systems, such as nanoparticles, could improve localization and reduce systemic toxicity. A clearer understanding of the context-specific functions of MMP-9 will guide the design of these new therapeutic strategies.

To move MMP-9 closer to clinical use as a treatment target, comprehensive and systematic clinical trials are needed to evaluate its efficacy, safety, and long-term effects. Additionally, given the variability among patients, precision medicine strategies based on MMP-9 could represent a major breakthrough in treating neuropathic pain. With rapid advancements in big data, artificial intelligence, and precision medicine, many studies now integrate genomics, proteomics, and clinical data to better understand MMP-9 and its pathways in various neuropathic pain types. This approach offers more precise evidence for personalized treatments (Knight et al., 2019; Osthues et al., 2020). Moreover, network pharmacology has identified new drug targets related to MMP-9, broadening the treatment options for neuropathic pain (Jo et al., 2023). However, these methods are still in early stages, and their clinical effectiveness has yet to be rigorously tested in large patient populations. The lack of standardized biomarkers for MMP-9 activity in human tissues makes it difficult to categorize patients or track therapeutic responses. Additionally, previous attempts to develop MMP inhibitors for cancer and inflammatory diseases failed in clinical trials due to poor specificity and adverse effects. These failures raise concerns about the safety and feasibility of MMP-9 inhibitors in managing chronic pain. Therefore, future clinical applications of MMP-9 inhibitors must be preceded by developing highly selective agents and robust diagnostic tools to guide targeted therapy.

In summary, MMP-9 plays a key role in the onset and progression of various types of peripheral neuropathic pain. Although its clinical application is still in early stages, growing clinical evidence supports its potential as both a biomarker and a therapeutic target. Importantly, clinical studies have begun to confirm mechanisms previously identified in preclinical models. For example, clinical data show that patients who experienced better postoperative analgesia after video-assisted thoracoscopic surgery lobectomy also had lower plasma MMP-9 levels, indicating its role in pain modulation (Chu et al., 2020). Other studies have found correlations between plasma MMP-9 levels and symptom severity in patients with acute coronary syndrome, suggesting that MMP-9 could be used as a biomarker for disease progression and patient stratification (Hamed and Fattah, 2015; Kobayashi et al., 2011). Although these studies do not yet demonstrate direct therapeutic modulation of MMP-9, they highlight its importance in human disease and support further investigation in precision medicine.

Taken together, these findings highlight the complex role of MMP-9 and other MMP isoforms in chronic pain. Targeted inhibition of specific MMP isoforms—particularly MMP-2 and MMP-9—has shown promise in reducing neuropathic pain while preserving normal matrix remodeling. However, challenges remain regarding specificity, pharmacokinetics, and off-target effects. Additionally, the potential of MMPs as diagnostic and prognostic biomarkers requires further validation, particularly for distinguishing between inflammatory and neuropathic pain. MMPs also modulate glial activation and neuroimmune interactions, making them key players in central sensitization and the persistence of chronic pain. Innovative techniques such as gene editing (e.g., CRISPR/Cas9) and RNA interference (e.g., siRNA, miRNA) offer promising approaches to selectively suppress MMP expression in targeted neural regions. These strategies could overcome the limitations of traditional small-molecule inhibitors. At the same time, understanding the interactions between MMPs and other pain-related pathways, such as Toll-like receptors, pro-inflammatory cytokines, and ion channels, may uncover additional targets for combination therapies. Future research should integrate these mechanistic insights with translational strategies, advancing MMP-targeted therapeutics that are not only effective but also safe and clinically viable. Ultimately, deepening our understanding of MMP biology in chronic pain will be essential in bridging the gap between bench research and clinical application.

## Glossary

AP-1: Activator protein 1
AQP4: Aquaporin 4
*β*-DG: Beta-dystroglycan
CX3CL1: C-X3-C motif chemokine ligand 1
CCI: Chronic constriction injury
CCI-IoN: Chronic constriction injury of the infraorbital nerve
CIPN: Chemotherapy-induced peripheral neuropathy
DRG: Dorsal root ganglion
ECM: Extracellular matrix
HMGB1: High-mobility group box 1
IASP: International Association for the Study of Pain
IDD: Intervertebral disk degeneration
IENF: Intraepidermal nerve fibers
IL-1β: Interleukin-1 beta
IL-6: Interleukin-6
LLLT: Low-level laser therapy
MMP-9: Matrix metalloproteinase-9
MMPs: Matrix metalloproteinases
NGF: Nerve growth factor
NMDAR: N-methyl-D-aspartate receptors
NR1: N-methyl-D-aspartate receptor subunit 1
NR2B: N-methyl-D-aspartate receptor subunit 2B
NSAIDs: Non-steroidal anti-inflammatory drugs
NF-κB: Nuclear factor kappa-light-chain-enhancer of activated B cells
PDN: Painful diabetic neuropathy
ROS: Reactive oxygen species
SASP: Senescence-associated secretory phenotype
SNC: Sciatic nerve crush
SNL: Spinal nerve ligation
TCAs: Tricyclic antidepressants
TIMPs: Tissue inhibitor of metalloproteinases
TN: Trigeminal neuralgia
TNF-*α*: Tumor necrosis factor-alpha
TRPV1: Transient receptor potential vanilloid 1
Nav1.7: Voltage-gated sodium channel subunit alpha-7
